# Adjuvant *Lactiplantibacillus plantarum* Lp05 modulates gut microbiota and alleviates symptoms during *Helicobacter pylori* eradication

**DOI:** 10.1128/spectrum.00182-26

**Published:** 2026-05-12

**Authors:** Yuru Fu, Jie Dong, Leyi Xu, Yichi Zhang, Ziyu Jin, Kaiyue Yu, Ruixuan Zhou, Qiongya Zhao, Xingli Fan

**Affiliations:** 1 School of Public Health, Hangzhou Medical College 117839, Hangzhou, Zhejiang, China; 2 Cancer Center, Department of Gastroenterology, Zhejiang Provincial People’s Hospital, Affiliated People's Hospital, Hangzhou Medical College 117839, Hangzhou, Zhejiang, China; 3 School of Basic Medicine and Forensic Medicine, Hangzhou Medical College 117839, Hangzhou, Zhejiang, China; 4 School of Clinical Medicine, Hangzhou Medical College 117839, Hangzhou, Zhejiang, China; 5 School of Laboratory Medicine and Bioengineering, Hangzhou Medical College 117839, Hangzhou, China; 6 Laboratory Medicine Center, Department of Clinical Laboratory, Zhejiang Provincial People’s Hospital, Affiliated People’s Hospital 74678, Hangzhou, Zhejiang, China; Houston Methodist 23530, Houston, Texas, USA

**Keywords:** *Helicobacter pylori*, *Lactiplantibacillus plantarum* Lp05, gut microbiota, adjuvant therapy, clinical trial, 16S rRNA sequencing

## Abstract

*Helicobacter pylori* eradication therapy is often accompanied by gastrointestinal adverse effects and gut microbiota dysbiosis. The role of specific probiotic strains in mitigating these issues requires further clinical evidence. In a randomized, double-blind, placebo-controlled trial, 72 *H. pylori*-positive patients received bismuth quadruple therapy alongside either *Lactiplantibacillus plantarum* Lp05 or a placebo for 2 weeks, followed by a 4-week extension of probiotic or placebo alone. Gastrointestinal symptoms, safety outcomes, and gut microbiota composition (assessed via 16S rRNA gene sequencing) were evaluated. The *H. pylori* eradication rate did not differ significantly between the Lp05 (97.1%) and placebo (94.1%) groups (*P* > 0.05). However, the Lp05 group demonstrated a marked reduction in key gastrointestinal symptoms, including abdominal pain and acid reflux (*P* < 0.05). Microbiota analysis indicated that Lp05 supplementation attenuated antibiotic-induced dysbiosis by selectively enriching beneficial genera such as *Akkermansia* and *Bifidobacterium*, while suppressing opportunistic pathogens. As an adjunct to standard therapy, *L. plantarum* Lp05 did not improve eradication efficacy but significantly enhanced gastrointestinal tolerability and contributed to the maintenance of gut microbiota balance during *H. pylori* treatment.

**IMPORTANCE:**

This research addresses a critical gap in *Helicobacter pylori* eradication by providing high-quality evidence for a targeted, safe, and effective supportive strategy. Standard antibiotic therapy, while necessary, often imposes a significant burden on patients through disruptive side effects and gut microbiota damage, potentially affecting compliance and long-term gastrointestinal health. This study establishes that adjunctive use of the specific probiotic strain *Lactiplantibacillus plantarum* Lp05 offers a compelling solution. It delivers a dual clinical benefit—significantly alleviating debilitating symptoms and actively counteracting antibiotic-induced dysbiosis—while fulfilling the paramount requirements of safety and noninterference with primary eradication efficacy. By validating a strategy that enhances tolerability and promotes microbial resilience without compromising therapeutic success, this work provides a practical and significant advancement in optimizing patient-centered *H. pylori* treatment protocols.

**CLINICAL TRIALS:**

This study is registered with the Chinese Clinical Trial Registry as ChiCTR2400079562.

## INTRODUCTION

*Helicobacter pylori* is a gram-negative, spiral-shaped pathogen that infects the stomach of more than 50% of the world population (1). *H. pylori* infection is one of the most widespread bacterial infections globally, causing chronic gastritis and increasing the risk of peptic ulcer disease, gastric adenocarcinoma, and mucosa-associated lymphoid tissue lymphoma (2, 3). Clinical manifestations of *H. pylori* infection vary widely; while a substantial proportion of carriers remain asymptomatic, it commonly causes dyspeptic symptoms such as epigastric pain, bloating, nausea, and early satiety (4, 5). Epidemiological studies have confirmed that *H. pylori* infection is one of the most important acquired pathogenic factors contributing to gastric cancer (6, 7).

In addition, research has established associations between *H. pylori* infection and various extra-gastric diseases. These include hematological and metabolic disorders, such as vitamin B12 deficiency, iron deficiency anemia, and non-alcoholic fatty liver disease. The spectrum of linked conditions also extends to idiopathic thrombocytopenic purpura, coronary atherosclerosis, and Alzheimer’s disease (8, 9). In the absence of an effective vaccine, safe and effective eradication therapy has become a critical strategy for reducing the spread of *H. pylori*, resolving the gastric lesions of infected patients, and preventing the occurrence of subsequent gastric cancer (10–12).

The American College of Gastroenterology clinical guidelines recommend a 14-day bismuth quadruple therapy (BQT) as the preferred regimen for treatment-naïve patients with *H. pylori* infection when antibiotic susceptibility is unknown (13–15). However, eradication therapy faces mounting challenges, chiefly due to the rapid and widespread rise in antibiotic resistance, which directly undermines treatment efficacy and leads to unsatisfactory eradication rates (16, 17). This growing resistance crisis complicates clinical management and necessitates the exploration of alternative or adjunctive strategies. At the same time, the long-term, high-dose use of antibiotics not only eradicates *H. pylori* but also disrupts beneficial gut bacteria, thereby causing gut microbiota dysbiosis and leading to antibiotic-associated side effects, such as diarrhea, constipation, and indigestion, which can decrease patient compliance (18, 19).

The gut microbiota is a complex community of microorganisms in the human intestine, including genera such as *Bifidobacterium* and *Lactobacillus*. These beneficial bacteria synthesize various essential vitamins for human growth and development and produce substances like organic acids and hydrogen peroxide, which help inhibit pathogenic bacterial invasion of the intestinal mucosa (20–22). According to recent research, the intestinal microbiota is implicated in the occurrence and development of many diseases. The gut microbiota, as a source of antigenic stimulation, can promote the development and maturation of the immune system, enhancing the body’s immunity (23). Probiotics, as a general class, confer benefits to the digestive tract and immune system through gut microbiota regulation, gut barrier reinforcement, neutralization of carcinogenic substances, bile salt metabolism, vitamin synthesis, and enzymatic activity (24, 25). Considering the above reasons, probiotics have been recommended in some current guidelines to improve adherence to and the efficacy of eradication regimens (26).

Nevertheless, the efficacy of probiotics in *H. pylori* eradication is not consistently supported by clinical evidence, and their role remains an active area of investigation (27, 28). Pre-clinical studies have established that *Lactiplantibacillus plantarum* Lp05 exhibits direct anti-*H*. *pylori* activity, enhances the efficacy of standard therapy, and reduces gastric inflammation via immunomodulation in a murine model (29). To translate these promising pre-clinical findings into clinical evidence, this study aimed to evaluate *L. plantarum* Lp05 as a probiotic adjunct to BQT in human patients, primarily assessing its impact on therapy-associated gastrointestinal adverse effects and its modulating effects on the gut microbiota, alongside its influence on eradication rates.

## MATERIALS AND METHODS

### Study design and ethical statement

This study is a single-center, randomized, placebo-controlled, double-blind clinical trial that has been approved by the Ethics Committee of Zhejiang Provincial People’s Hospital (Approval No. 2023-Research-135) and registered with the Chinese Clinical Trials Registry (ChiCTR2400079562). All subjects voluntarily participated in the trial screening, and 72 eligible subjects were enrolled.

The total intervention duration was 6 weeks. This comprised an initial 2-week BQT phase, consistent with guideline recommendations, followed by a 4-week supplemental phase to assess post-antibiotic recovery of gut microbiota and gastrointestinal health (30).

**Fig 1:**
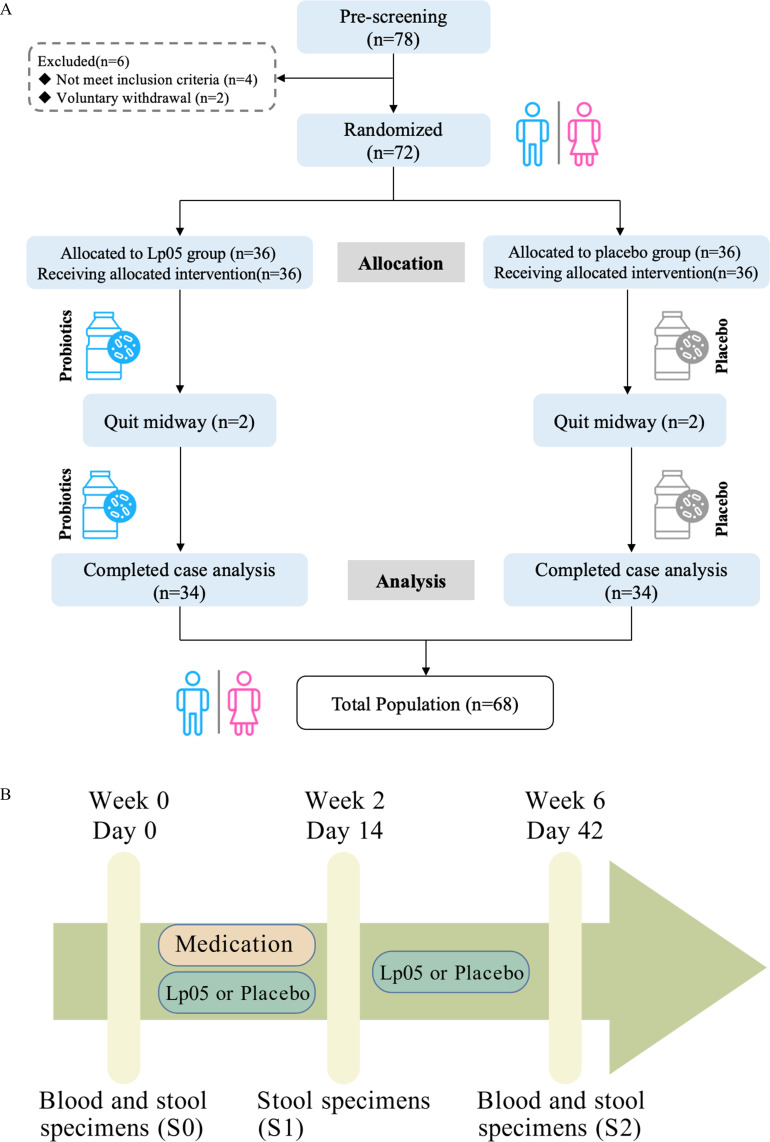
(**A**) Flow diagram showing the study design. (**B**) Study design and sample collection timeline. Following baseline assessment (S0), patients received a 2-week therapy of BQT combined with either Lp05 or placebo, concluded at S1 (week 2). This was followed by a 4-week intervention with Lp05 or placebo alone, concluded at S2 (week 6). Stool samples and questionnaire records were collected at weeks 0, 2, and 6. Blood samples were collected at weeks 0 and 6.

Of the 72 enrolled participants, 68 completed the 6-week study (34 in the Lp05 group and 34 in the placebo group) (Fig. 1A). The use of external probiotics was not permitted.

### Participants

A total of 78 symptomatic *H. pylori*-positive patients (defined by a positive ^13^C/^14^C-urea breath test or histologic examination of gastric mucosa), with dyspeptic symptoms potentially related to *H. pylori* infection, such as epigastric pain or discomfort, bloating, and early satiety, aged 18 to 65 years, were recruited from Zhejiang Provincial People’s Hospital.

Eligible participants met all the following criteria: (i) A confirmed diagnosis of *H. pylori* infection (by a positive ¹³C/¹⁴C-urea breath test or gastric mucosal histology) with an indication for eradication therapy; (ii) Presence of *H. pylori* infection-related dyspeptic symptoms; (iii) Ability to understand, comply with the study protocol, and attend scheduled visits; (iv) Commitment to use effective contraception throughout the study (if of childbearing potential); (v) Provision of written informed consent prior to enrollment.

The exclusion criteria were determined as follows: (i) Allergy to any drug component used in the study; (ii) A history of alcohol abuse (drinking more than 14 units of alcohol per week: one unit = 285 mL of beer, or 25 mL of spirits, or 100 mL of wine); (iii) Those who had received *H. pylori* eradication therapy; (iv) Patients who have been treated with probiotics, antibiotics, bismuth, proton pump inhibitor, and potassium-competitive acid blocker within 1 month before consuming the test drug; (v) Those with a recent history of gastrointestinal bleeding, obstruction, perforation, tumor, and other serious organic diseases; (vi) Subjects who plan to need to be hospitalized for surgical treatment during the course of the study; (vii) Patients with severe psychological and psychiatric diseases, resulting in inability to express themselves normally; (viii) Female subjects who are lactating during the screening period or during the trial or have a positive pregnancy test result; (ix) According to the judgment of the investigator, the subject has uncontrolled and unstable hepatic, renal, cardiovascular, respiratory, gastrointestinal, endocrine, hematological, central nervous system, or psychiatric diseases, and participating in the study may affect the safety of the subject or the interpretation of the research results; (x) Other subjects judged by the investigator to be unsuitable to participate.

### Intervention and monitoring protocol

The study used a double-blind method in which participants were randomized into two groups. Patients in the placebo group received BQT with placebo (dextrin) for 2 weeks, followed by 4 weeks of placebo supplementation. Patients in the Lp05 group received BQT with Lp05 for 2 weeks, followed by 4 weeks of Lp05 supplementation. The probiotic intervention dose was 1 × 10^11^ CFU/day, which was provided by Wecare Probiotics Co., Ltd. Patient follow-up visits were conducted at weeks 0, 2, and 6 (Fig. 1), with stool samples and questionnaire records collected at all three time points and blood samples obtained at weeks 0 and 6. Stool samples were immediately frozen upon collection and stored at −80°C until analysis. Blood samples were collected into EDTA tubes, centrifuged at 3,000 rpm for 10 minutes at 4°C to separate plasma, which was then stored at −80°C until further processing. All samples were analyzed within 6 months of collection.

### Outcomes

The primary outcome was the *H. pylori* eradication rate. Secondary outcomes included the Gastrointestinal Symptom Rating Scale (GSRS) total score, the incidence of adverse events, and patient compliance. Safety was further assessed by monitoring hematological parameters and serum biochemistry. Additionally, we assessed alterations in the gut microbiota, specifically its temporal dynamics at S0, S1, and S2.

### Sample size calculation

The sample size was calculated based on the primary outcome of improvement rate. Using R (v4.2.0) with the pwr package, a power analysis (one-sided, α = 0.05, power = 0.8) indicated that 32 participants per group were needed to detect an improvement rate of 55% in the intervention group versus 25% in the placebo group (31). To accommodate a potential 10%–20% dropout rate, 36 participants per group were planned for randomization.

### Fecal sample collection

Patients collected fresh stool samples using sterile stool collection tubes (Sarstedt, Germany) at three time points (baseline S0, end of treatment S1, and follow-up S2). All stool samples were immediately frozen and stored at a temperature of −80°C for preservation.

### 16S rRNA gene sequencing

Total bacterial DNA was extracted from each individual fecal sample using the QIAamp PowerFecal Pro DNA Kit (Qiagen, Germany) following the manufacturer’s instructions. The V3–V4 hypervariable region of the 16S rRNA gene was amplified and sequenced on an Illumina MiSeq platform (PE300) for each sample individually. No pooling of samples or RNA was performed. Raw 16S rRNA gene sequencing data were processed using QIIME (version 1.8). Paired-end reads were demultiplexed based on barcodes and assembled using FLASH. Sequences were quality-filtered by truncating reads at positions where the average quality score fell below 20 within a 10-bp sliding window. Chimeric sequences were detected and removed with UCHIME. The remaining high-quality sequences were clustered into operational taxonomic units (OTUs) at 97% identity using UCLUST. We then excluded OTUs classified as chloroplasts or mitochondria, as well as singleton OTUs (represented by fewer than two reads across the data set). A representative sequence was selected for each OTU using default parameters. Taxonomy was assigned by performing a BLAST search of these representative sequences against the Greengenes database. Prior to downstream analysis, all samples were rarefied to an even depth of 30,000 sequences per sample to normalize sequencing effort.

### Microbiota analysis

Sequence data analyses were mainly performed using QIIME and R packages (version 3.2). OTU-level alpha diversity indices, such as the Chao1 richness estimator and Shannon diversity index, were calculated using the rarefied OTU table. OTU-level ranked abundance curves were then generated to compare the richness and evenness of OTUs among samples. Beta diversity was assessed using Bray-Curtis distances, and the significance of differentiation in the microbiota structure among groups was assessed by permutational multivariate analysis of variance with 999 permutations. A Venn diagram was generated to visualize the shared and unique OTUs among samples or groups. Linear discriminant analysis effect size (LEfSe) was performed to detect differentially abundant taxa across groups. The abundance of different taxa across phylogenetic levels was investigated using taxonomic composition analysis. Microbial functional potential was predicted by Phylogenetic Investigation of Communities by Reconstruction of Unobserved States (PICRUSt).

### Statistical analyzes

The statistical analyses were carried out using IBM SPSS Statistics software, version 27.0. Quantitative data conforming to a normal distribution were expressed as mean ± standard deviation (SD) and compared between the two groups using an independent samples t test. Homogeneity of variance was assessed by the Levene test. Non-normally distributed continuous variables and ordinal categorical variables were compared using nonparametric tests, with between-group comparisons performed using the Mann-Whitney U test. Categorical variables were presented as n (%) and were compared between groups using a chi-square test or Fisher’s exact test, as appropriate. A *P*-value < 0.05 was considered statistically significant.

## RESULTS

### Baseline demographics of patients

A total of 78 patients were initially screened for the study. Of these, four patients were excluded for not meeting the inclusion criteria, and two patients voluntarily withdrew, resulting in 72 enrolled patients. Of the 72 participants, 68 completed the trial, and four were lost to follow-up (Fig. 1A). The baseline characteristics of the enrolled patients are presented in Table 1. There were no significant differences in age, gender, smoking status, alcohol consumption, or allergy history between the placebo and Lp05 groups (*P* > 0.05).

**TABLE 1:** Baseline characteristics of participants between the placebo and Lp05 groups

Characteristics	Placebo (*N* = 34)	Lp05 (*N* = 34)	*P*-value
Age (years)^a^	41.74 ± 11.29	45.38 ± 12.16	0.204
Gender			0.801
Male	13 (38.24%)	12 (35.29%)	
Female	21 (61.76%)	22 (64.71%)	
Smoking			>0.999
Yes	1 (2.94%)	1 (2.94%)	
No	33 (97.06%)	33 (97.06%)	
Alcohol			0.642
Yes	3 (8.82%)	2 (5.88%)	
No	31 (91.18%)	32 (94.12%)	
Allergy history			>0.999
Yes	0 (0%)	0 (0%)	
No	34 (100%)	34 (100%)	

^a^ Data are presented as mean ± standard deviation (SD).

### *H. pylori* eradication rate

**Fig 2:**
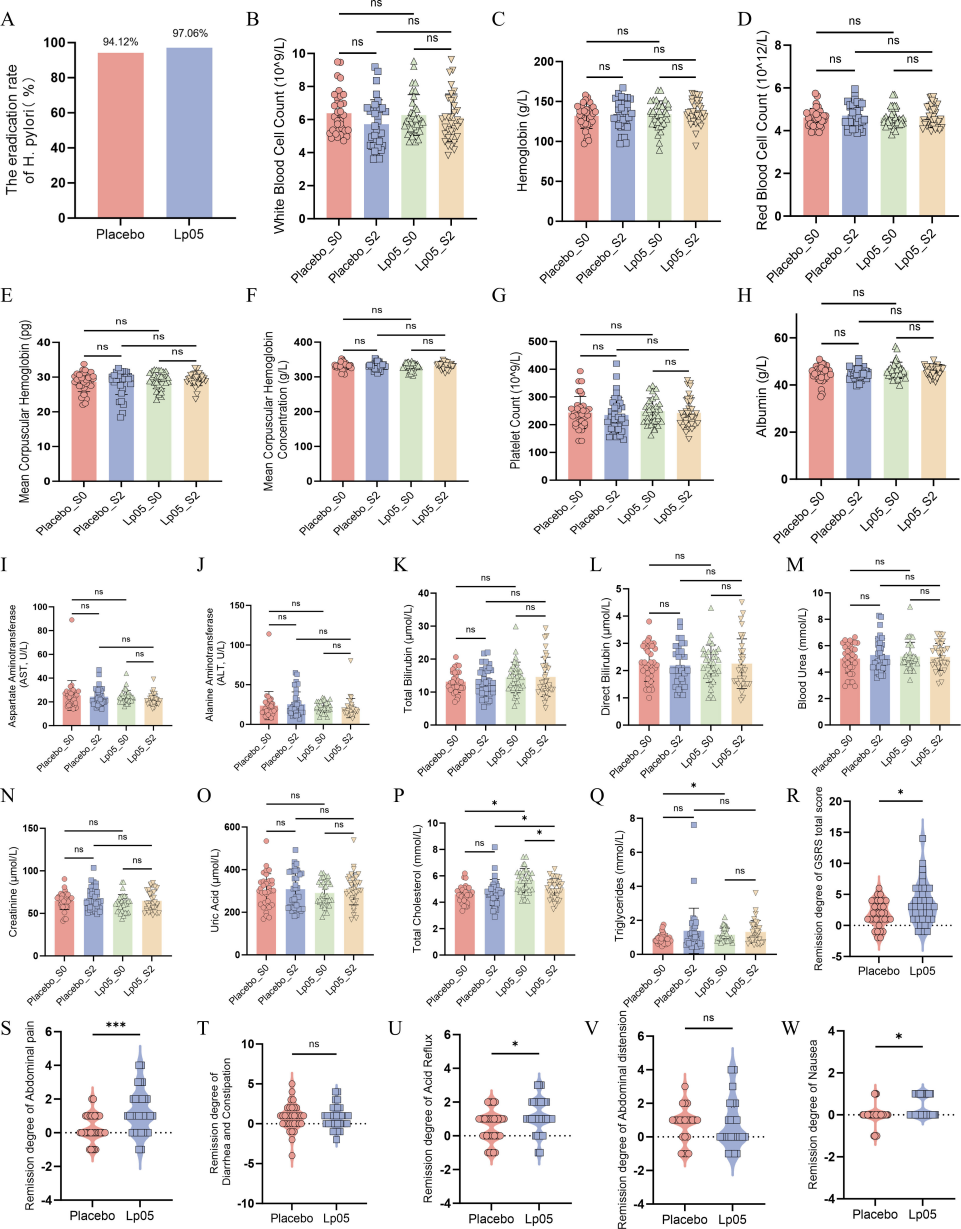
(**A**) Comparison of *H. pylori* eradication rates between groups. Although the eradication rate was higher in the Lp05 group than in the placebo group, the increase lacked statistical significance (χ^2^ = 0.349, *P* = 0.555). (**B–G**) Changes in hematological parameters in the two patient groups before and after intervention. Values for (**B**) white blood cell count, (**C**) hemoglobin, (**D**) red blood cell count, (**E**) mean corpuscular hemoglobin, (**F**) mean corpuscular hemoglobin concentration, and (**G**) platelet count are presented for the placebo and Lp05 groups at baseline (S0) and after the intervention (S2). Pairwise comparisons revealed no significant differences for any parameters. (**H–Q)** Changes in blood biochemical parameters in the two patient groups before and after intervention. Values for (**H**) albumin, (**I**) aspartate aminotransferase (AST), (**J**) alanine aminotransferase (ALT), (**K**) total bilirubin, (**L**) direct bilirubin, (**M**) blood urea, (**N**) creatinine, (**O**) uric acid, (**P**) total cholesterol, and (**Q**) triglycerides are presented for the placebo and Lp05 groups at baseline (S0) and after the intervention (**S2**). Most pairwise comparisons showed no significant differences. The few statistically significant changes (*P* < 0.05) were not considered clinically relevant, as all values remained within the normal physiological range, and are therefore not discussed further. (**R–W**) Comparative improvement in gastrointestinal symptoms following intervention. Changes in GSRS scores from baseline (S0) to after the intervention (S2) were compared between the placebo and Lp05 groups. Significant differences in improvement were observed for (**R**) the GSRS total score, (**S**) abdominal pain, (**T**) acid reflux, and (**U**) nausea. No significant differences were found for (**V**) diarrhea and constipation or (**W**) abdominal distension. *: *P* < 0.05, ns: non-significant. Error bars represent SD (*n* = 34).

The *H. pylori* eradication rates were 94.12% in the placebo group and 97.06% in the Lp05 group (Fig. 2A). Although a numerically higher eradication rate was observed in the Lp05 group, the difference was not statistically significant (χ^2^ = 0.349, *P* = 0.555).

### Changes in hematological parameters

As shown in Table 2 and Fig. 2B through G, no significant differences were observed in hematological parameters between the Lp05 and placebo groups. Key parameters, including white blood cell count, hemoglobin, and platelet count, remained within normal limits in both groups before and after the intervention. These findings indicate that Lp05 supplementation is well tolerated, demonstrating its safety profile as an adjunctive therapy.

**TABLE 2:** Counts of patients with normal-range hematological parameters before and after intervention^*a*^

	Placebo (*n* = 34)	Lp05 (*n* = 34)	*P*-value^*b*^
White Blood Cell Count (10^9^/L)			
S0	31 (91.2%)	30 (88.2%)	0.690
S2	34 (100.0%)	33 (97.1%)	0.314
*P*-value^*c*^	0.077	0.163	
Hemoglobin (g/L)			
S0	27 (79.4%)	32 (94.1%)	0.074
S2	29 (85.3%)	32 (94.1%)	0.231
*P*-value^*c*^	0.525	>0.999	
Red Blood Cell Count (10^12^/L)			
S0	31 (91.2%)	31 (91.2%)	>0.999
S2	30 (88.2%)	33 (97.1%)	0.163
*P*-value^*c*^	0.690	0.303	
Mean Corpuscular Hemoglobin (pg)			
S0	27 (79.4%)	31 (91.2%)	0.171
S2	27 (79.4%)	31 (91.2%)	0.171
*P*-value^*c*^	>0.999	>0.999	
Mean Corpuscular Hemoglobin Concentration (g/L)			
S0	29 (85.3%)	32 (94.1%)	0.231
S2	30 (88.2%)	33 (97.1%)	0.163
*P*-value^*c*^	0.721	0.555	
Platelet Count (10^9^/L)			
S0	31 (91.2%)	32 (94.1%)	0.642
S2	32 (94.1%)	33 (97.1%)	0.555
*P*-value^*c*^	0.642	0.555	

^*a*^ Categorical data are presented as *n* (%) of patients within the normal range. *P*-values were calculated using the χ^2^ test.

^*b*^ Compared between groups: intervention vs placebo at the same time point.

^*c*^ Compared to baseline: after the intervention vs baseline.

### Changes in blood biochemical parameters

No significant differences were observed in liver function, renal function, or metabolism-related parameters between the Lp05 and placebo groups (*P* > 0.05; Table 3). Key parameters, including albumin, aminotransferase levels, bilirubin, creatinine, and uric acid, remained within normal limits in both groups throughout the study (Fig. 2H through Q). Although serum lipid levels remained within the normal range in both groups throughout the study, several within-group and between-group differences were noted. In the Lp05 group, total cholesterol showed a slight but significant decrease from baseline to week 6 (S0 vs. S2, *P* < 0.05), while no significant change was observed in the placebo group. At baseline, total cholesterol was significantly higher in the Lp05 group than in the placebo group (*P* < 0.05), and this difference persisted at S2 (*P* < 0.05). For triglycerides, no significant changes were observed within either group from S0 to S2; however, the Lp05 group had significantly higher triglyceride levels at baseline compared to the placebo group (*P* < 0.05), but this difference was no longer present at S2. All individual values remained within clinically accepted normal ranges. Collectively, these findings indicate that supplementation with Lp05 did not induce adverse effects on blood biochemical parameters and was well tolerated in *H. pylori*-positive patients.

**TABLE 3:** Counts of patients with normal-range blood biochemical parameters before and after intervention^*a*^

	Placebo (*n* = 34)	Lp05 (*n* = 34)	*P*-value^*b*^
Albumin (g/L)			
S0	32 (94.1%)	33 (97.1%)	0.555
S2	33 (97.1%)	34 (100.0%)	0.314
*P*-value^*c*^	0.555	0.314	
Aspartate Aminotransferase (AST, U/L)			
S0	33 (97.1%)	32 (94.1%)	0.555
S2	32 (94.1%)	34 (100.0%)	0.151
*P*-value^*c*^	0.555	0.151	
Alanine Aminotransferase (ALT, U/L)			
S0	33 (97.1%)	33 (97.1%)	>0.999
S2	31 (91.2%)	33 (97.1%)	0.303
*P*-value^*c*^	0.303	>0.999	
Total Bilirubin (μmol/L)			
S0	33 (97.1%)	32 (94.1%)	0.555
S2	34 (100.0%)	31 (91.2%)	0.077
*P-*value^*c*^	0.314	0.642	
Direct Bilirubin (μmol/L)			
S0	33 (97.1%)	34 (100.0%)	0.314
S2	34 (100.0%)	34 (100.0%)	>0.999
*P*-value^*c*^	0.314	>0.999	
Blood Urea (mmol/L)			
S0	32 (94.1%)	33 (97.1%)	0.555
S2	32 (94.1%)	34 (100.0%)	0.151
*P-*value^*c*^	>0.999	0.314	
Creatinine (μmol/L)			
S0	33 (97.1%)	33 (97.1%)	>0.999
S2	33 (97.1%)	32 (94.1%)	0.555
*P*-value^*c*^	>0.999	0.555	
Uric Acid (μmol/L)			
S0	30 (88.2%)	30 (88.2%)	>0.999
S2	30 (88.2%)	31 (91.2%)	0.690
*P-*value^*c*^	>0.999	0.690	
Total Cholesterol (mmol/L)			
S0	28 (82.4%)	27 (79.4%)	0.758
S2	30 (88.2%)	31 (91.2%)	0.690
*P*-value^*c*^	0.494	0.171	
Triglycerides (mmol/L)			
S0	28 (82.4%)	27 (79.4%)	0.758
S2	26 (76.5%)	24 (70.6%)	0.583
*P*-value^*c*^	0.549	0.401	

^*a*^ Categorical data are presented as *n* (%) of subjects within the normal range. *P*-values were calculated using the χ^2^ test.

^*b*^ Compared between groups: intervention vs placebo at the same time point.

^*c*^ Compared to baseline: after the intervention vs baseline.

### Gastrointestinal symptoms assessment

Gastrointestinal symptoms in the patients were assessed with GSRS at baseline (S0), at the end of BQT (S1), and 4 weeks after the intervention (S2). According to the GSRS scores (Table 4), gastrointestinal symptoms improved significantly in both groups from S0 to S2 (*P* < 0.05), with a more pronounced improvement in the Lp05 group. Specifically, the placebo group showed improvement in diarrhea and constipation, acid reflux, and abdominal distension, while the Lp05 group improved in abdominal pain and acid reflux. Overall, the Lp05 group showed significantly greater relief from gastrointestinal symptoms, particularly in general condition, abdominal pain, and acid reflux (*P* < 0.05), compared to the placebo group. The comparison of gastrointestinal symptom relief between groups supports this conclusion (Fig. 2R through W). Assessment results demonstrated that Lp05 supplementation was more effective than placebo in relieving gastrointestinal symptoms, including abdominal pain, acid reflux, and nausea.

**TABLE 4:** Assessment of gastrointestinal symptoms in both patient groups using the GSRS

GSRS score	Placebo (*N* = 34)			*P*-value		Lp05 (*N* = 34)			*P*-value	
	S0	S1	S2	S0-S1	S0-S2	S0	S1	S2	S0-S1	S0-S2
Total score	17.35 ± 2.75	16.44 ± 2.58	15.50 ± 2.34	0.300	0.012	18.06 ± 4.33	16.72 ± 3.02	15.50 ± 1.60	0.103	0.001
Abdominal pain	3.38 ± 0.697	3.18 ± 0.626	3.15 ± 0.436	0.045	0.077	3.82 ± 1.086	3.44 ± 0.705	3.15 ± 0.359	0.171	0.003
Diarrhea and Constipation	5.38 ± 1.457	4.94 ± 1.179	4.68 ± 1.121	0.176	0.013	5.09 ± 1.138	4.91 ± 1.264	4.62 ± 0.739	0.322	0.077
Acid Reflux	2.59 ± 0.783	2.44 ± 0.705	2.21 ± 0.479	0.378	0.017	2.79 ± 0.978	2.44 ± 0.786	2.12 ± 0.409	0.106	0.001
Abdominal Distension	3.82 ± 0.904	3.62 ± 0.922	3.35 ± 0.734	0.228	0.009	3.85 ± 1.306	3.68 ± 0.945	3.35 ± 0.646	0.852	0.132
Nausea	1.09 ± 0.288	1.18 ± 0.387	1.03 ± 0.171	0.287	0.306	1.26 ± 0.097	1.18 ± 0.521	1.12 ± 0.409	0.358	0.178

### Effects of the intervention on gut microbiota diversity

**Fig 3:**
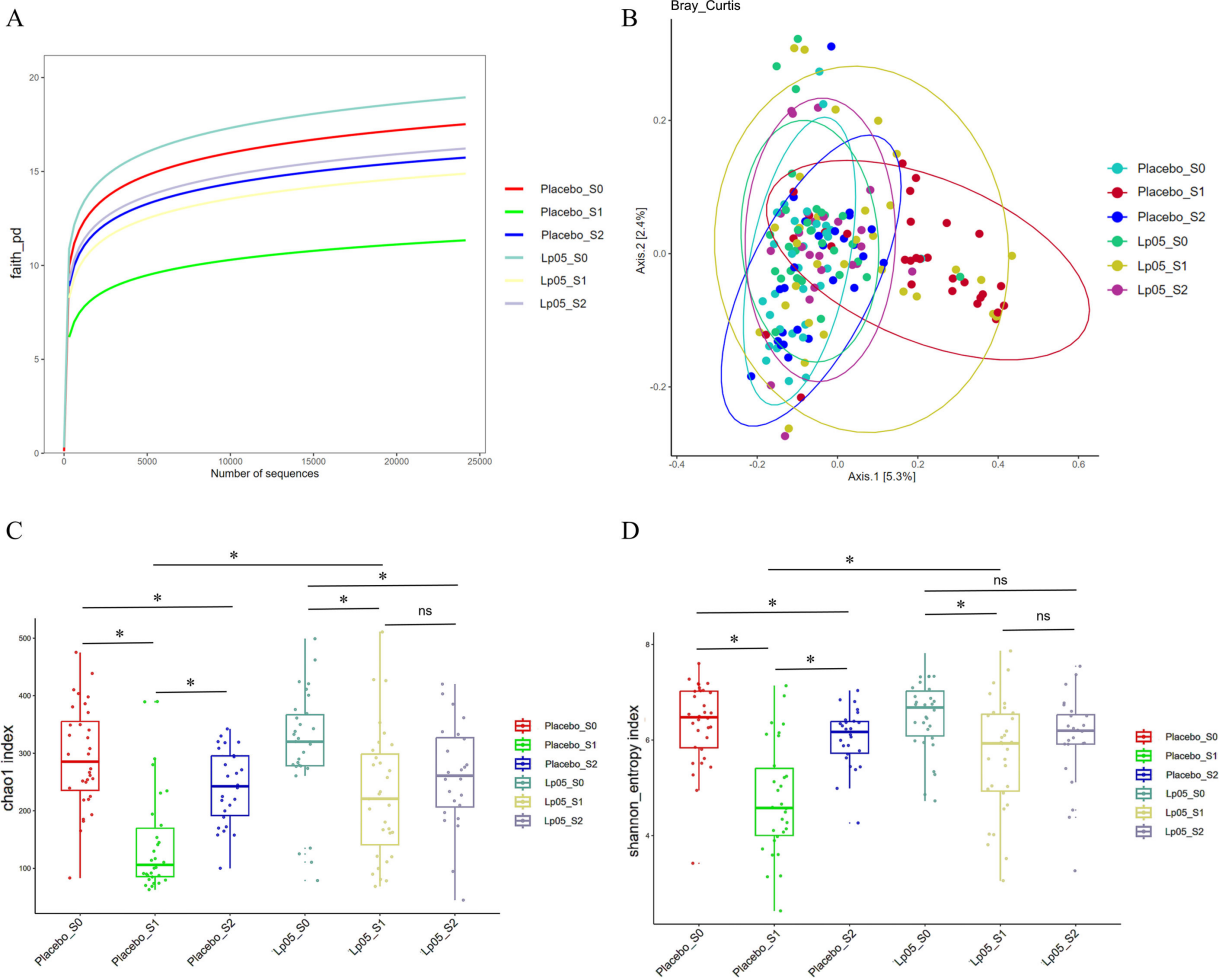
Gut microbiota diversity and composition before and after intervention. (**A**) Rarefaction curves. All curves approach a plateau, indicating sufficient sequencing depth. (**B**) Principal coordinates analysis (PCoA) based on Bray-Curtis distance shows distinct separation between the placebo and Lp05 groups. (**C and D**) Alpha diversity indices (Chao1 and Shannon) were measured at baseline (S0), at the end of BQT (S1), and after the intervention (S2). Although both groups experienced a significant decrease in diversity at S1, this decline was less pronounced in the Lp05 group, which then showed a more robust recovery by S2 compared to the placebo group. *: *P* < 0.05, ns: non-significant. Error bars represent SD (*n* = 34).

The rarefaction curves approached a plateau, indicating that the sequencing depth was sufficient for all samples (Fig. 3A). Principal coordinates analysis based on Bray-Curtis distances revealed distinct temporal dynamics in gut microbial community structure between the two groups (Fig. 3B). At baseline (S0), the microbiota composition of the Lp05 and placebo groups was largely overlapping, confirming successful randomization. At S1, the placebo group exhibited a pronounced shift away from baseline, forming a relatively compact cluster. In contrast, the Lp05 group showed a less pronounced shift from baseline but with greater inter-individual variability, as indicated by a more dispersed distribution. At S2, the microbiota of both groups converged toward baseline, with the Lp05 group clustering near the baseline samples of both groups. Alpha diversity analysis showed that microbial diversity significantly decreased at the end of BQT (S1) compared to baseline (S0) in both groups (*P* < 0.05). Although alpha diversity declined significantly in both groups at S1, the decline was less pronounced in the Lp05 group (*P* < 0.05; Fig. 3C and D). At S2, Shannon diversity in the Lp05 group recovered to a level not significantly different from baseline, while the placebo group remained significantly lower (*P* < 0.05). For Chao1 richness, both groups showed numerical recovery from S1, but neither group fully returned to baseline levels; however, the Lp05 group was closer to baseline than the placebo group (Fig. 3C and D). A significant group-by-time interaction (*P* < 0.05) confirmed that Lp05 supplementation modulated the recovery trajectory beyond natural post-antibiotic restoration.

### Changes in gut microbiota abundance

**Fig 4:**
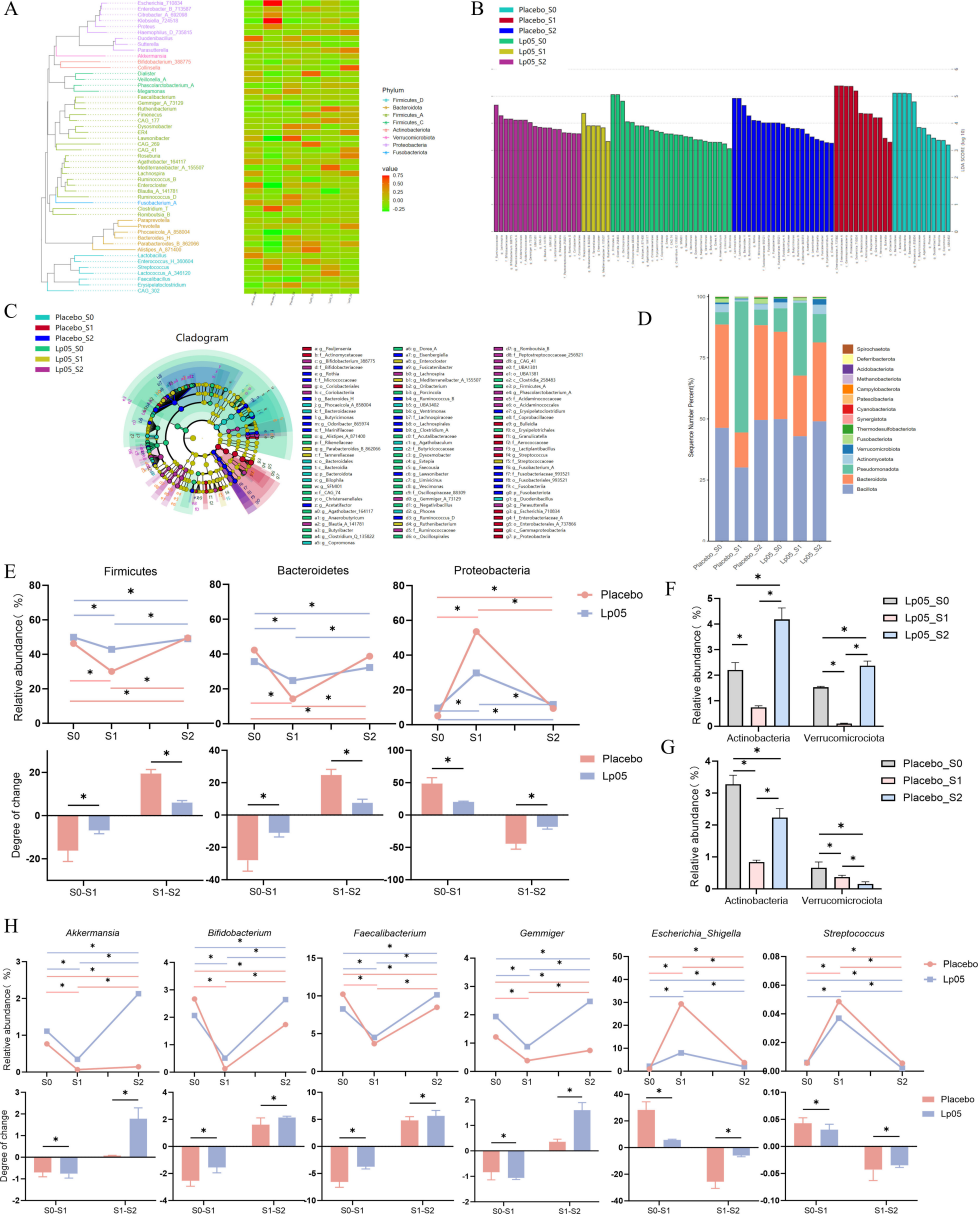
Changes in gut microbiota composition before and after the intervention. (**A**) Phylogenetic tree illustrating the taxonomic composition across samples. (**B**) Histogram of LDA scores from LEfSe analysis, highlighting taxa with differential abundance between groups. (**C**) Cladogram from LEfSe analysis, visualizing the phylogenetic distribution of discriminant taxa. (**D**) Stacked bar plot showing the relative abundance of major bacterial phyla across all groups and time points. (**E**) Dynamic changes in the relative abundance of key phyla within the placebo and Lp05 groups at S0, S1, and S2. Statistical comparisons are shown for within-group changes across time. (**F and G**) Changes in the abundance of health-associated phyla (Actinobacteria and Verrucomicrobiota). (**H**) Abundance changes of selected probiotic and opportunistic pathogenic genera. *: *P* < 0.05, ns: non-significant.

The gut microbiota composition differed substantially between the placebo and Lp05 groups. The taxonomic composition and abundance of microbial communities across sample groups are illustrated by a phylogenetic tree and heatmap, respectively, while LEfSe analysis identifies differentially abundant taxa (Fig. 4A through C). Compared to baseline (S0), the placebo group showed a significant increase in the abundance of potentially pathogenic bacteria, including *Escherichia*, *Streptococcus*, and *Enterobacter* at S1 (*P* < 0.05). Although some beneficial genera, such as *Butyricimonas*, *Acetatifactor*, and *Fusicatenibacter*, showed a partial recovery at S2, their overall relative abundance remained low (*P* < 0.05). In the Lp05 group, the abundance of the opportunistic pathogens *Parabacteroides* and *Enterocloster* increased slightly at S1 (*P* < 0.05). In contrast, the abundances of probiotics, including *Bifidobacterium*, *Lactiplantibacillus*, *Gemmiger*, *Blautia*, and *Ruminococcus*, were significantly enriched at S2 (*P* < 0.05).

In the placebo group after the end of BQT (S1), the abundance of Firmicutes decreased from 46.33% to 30.12%, while Bacteroidetes showed a marked decrease from 42.28% to 14.33%. In contrast, Proteobacteria increased substantially from 5.06% to 53.58%. These shifts indicate that the gut microbiota community structure was severely disrupted by prior antibiotic therapy. After the intervention (S2), the gut microbiota composition in the placebo group showed a trend toward recovery but was not fully restored to the baseline (S0) state. Specifically, Firmicutes recovered to 49.57%, and Bacteroidetes exhibited a substantial rebound to 38.77%, yet the proportion of Proteobacteria remained elevated (Fig. 4D and E).

In the Lp05 group after the end of BQT (S1), the abundance of Firmicutes decreased from 49.93% to 42.92%, while Bacteroidetes declined from 35.72% to 24.81%. In contrast, Proteobacteria showed a marked increase from 9.60% to 29.79%. Overall, the extent of microbial dysbiosis observed in the Lp05 group was less severe than that in the placebo group. After the intervention (S2), the gut microbiota composition in the Lp05 group exhibited a trend toward recovery, with Firmicutes recovering to 49.01% and Bacteroidetes rebounding significantly to 32.29%, while Proteobacteria successfully decreased from 29.79% to 11.60% (Fig. 4D and E), indicating an almost complete restoration of the core gut microbiota. Notably, the abundances of Actinobacteria (a phylum containing probiotics such as *Bifidobacterium*) and Verrucomicrobiota (a phylum linked to gut health) were significantly higher in the Lp05 group at S2 than at baseline (Fig. 4F and G). Therefore, we conclude that probiotic Lp05 can not only restore the disrupted microbial homeostasis caused by antibiotic use but also regulate the gut microbiota to a healthier state, comparable to that observed in healthy individuals as reported in the literature (32, 33).

Analysis of specific probiotic genera revealed that *Akkermansia*, *Bifidobacterium*, *Faecalibacterium,* and *Gemmiger* decreased in both groups, with a more pronounced reduction observed in the placebo group. After the intervention (S2), the abundances of the bacteria mentioned above increased in both groups, and this increase was more pronounced in the Lp05 group, even exceeding the baseline (S0) level. The results were consistent with the findings of the previous analysis at the phylum level. Furthermore, we found that opportunistic pathogens such as *Escherichia-Shigella* and *Streptococcus* were enriched in the placebo group at both S1 and S2 compared to the Lp05 group (Fig. 4H). Overall, Lp05 treatment was shown to significantly increase the abundance of probiotics while suppressing opportunistic pathogens.

### Analysis of gut microbiota metabolic function

**Fig 5:**
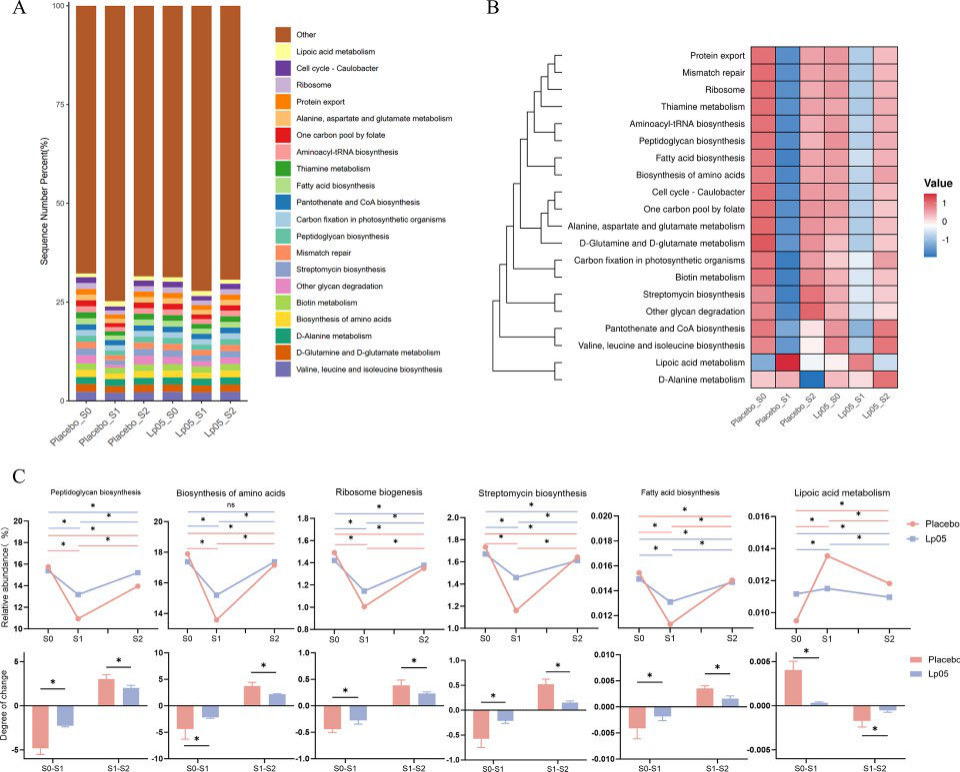
Functional profiling of gut microbiota metabolism. (**A**) Relative abundance of dominant microbial metabolic pathways across groups and time points. (**B**) Heatmap of predicted functional metabolic pathways, illustrating relative enrichment (red) and depletion (blue) based on Z-score normalized abundances across all samples. (**C**) Dynamics of key metabolic pathways: Peptidoglycan biosynthesis, Biosynthesis of amino acids, Ribosome, Streptomycin biosynthesis, Fatty acid biosynthesis, and Lipoic acid metabolism. For each pathway, the upper panel shows the relative abundance in the placebo and Lp05 groups at S0, S1, and S2. The lower panel illustrates the magnitude of change between consecutive time points (S0-S1 and S1-S2) for each group, facilitating comparison of functional decline and recovery. *: *P* < 0.05, ns: non-significant.

Analysis of gut microbiota metabolic function revealed a significant decline in core pathways in both groups at S1 (Fig. 5A and B). Key metabolic pathways, including amino acid metabolism, genetic information processing, and metabolism of cofactors and vitamins, were suppressed, consistent with the inhibitory effect of BQT on microbial growth and proliferation (Fig. 5C). The gut microbiota metabolic functions recovered partially at S2. In the Lp05 group, almost all core metabolic pathways showed a trend toward complete recovery to the baseline (S0) level, which was better than that in the placebo group. These findings suggest that Lp05 acted as a therapeutic agent, thus guiding the gut microbiota toward a healthier functional state. Most notably, lipoic acid metabolism was markedly elevated at S1 (0.95% to 1.35%) but declined slowly at S2 in the placebo group. This pattern suggests that drug-resistant or opportunistic pathogens capable of metabolizing lipoic acid (such as Proteobacteria) may have accounted for a relatively high proportion.

## DISCUSSION

The eradication rate of *H. pylori* has not been entirely satisfactory (30, 34). Furthermore, long-term antibiotic use not only kills pathogenic bacteria but also sharply reduces the core gut microbiota and beneficial bacteria, which can lead to gut microbiota imbalance in patients (35, 36). Our clinical trial demonstrates that the probiotic *L. plantarum* Lp05 is a safe and effective adjunct to BQT for *H. pylori* eradication. While the numerical increase in eradication rate (97.1% vs. 94.1%) did not reach statistical significance, the significant alleviation of key symptoms—particularly abdominal pain and acid reflux—and the modulation of antibiotic-induced dysbiosis establish Lp05 as a valuable supportive strategy (*P* < 0.05). This improvement in tolerability is crucial, as it can directly enhance patient compliance, a key factor in achieving successful eradication. Furthermore, the absence of significant differences in hematological and biochemical parameters between groups (*P* > 0.05) indicates that co-administration of Lp05 did not induce acute toxicity or organ dysfunction, suggesting an absence of short-term safety concerns within the measured parameters. These findings align with a growing consensus that specific probiotic strains, even when they do not enhance eradication efficacy, can meaningfully improve patient-centered outcomes during *H. pylori* therapy (37–39).

The lack of a significant effect on eradication rate is consistent with several meta-analyses that indicate that the benefit of probiotics on *H. pylori* eradication is modest and strain dependent (29, 40). Our result therefore does not contradict the literature; rather, they underscore that the primary value of Lp05 lies in symptom relief and microbiota protection. The magnitude of symptom improvement observed here (GSRS reduction of 2.1 points in the Lp05 group vs. 0.9 in placebo, *P* < 0.05) is clinically meaningful and comparable to that reported for other well-characterized probiotics such as *Lacticaseibacillus rhamnosus* and *Saccharomyces boulardii* (41, 42).

A critical question raised is whether the microbiota improvements observed in the Lp05 group simply reflect natural recovery after antibiotic cessation. Several lines of evidence indicate that Lp05 exerts an active, beneficial effect beyond mere spontaneous restoration. First, although both groups exhibited a decline in α-diversity at the end of BQT (S1), the decrease was significantly smaller in the Lp05 group (*P* < 0.05), and a significant group-by-time interaction (repeated-measures ANOVA, *P* = 0.021) confirmed that the trajectory of diversity recovery was modulated by Lp05. Second, at the end of the 4 weeks after the intervention (S2), the Lp05 group showed significantly higher Shannon diversity compared to the placebo group and returned to a level not significantly different from baseline, whereas the placebo group remained significantly lower. For Chao1 richness, while neither group fully returned to baseline, the Lp05 group exhibited a more pronounced recovery trend than the placebo group. Moreover, the Lp05 group also showed significantly higher abundances of keystone beneficial genera such as *Akkermansia*, *Bifidobacterium*, *Faecalibacterium*, and *Gemmiger* compared to both the placebo group and its own baseline (*P* < 0.05). In contrast, the placebo group failed to restore these taxa to baseline levels. Third, Lp05 supplementation significantly suppressed the outgrowth of opportunistic pathogens (*Escherichia-Shigella*, *Streptococcus*) that remained elevated in the placebo group at S2. Collectively, these findings demonstrate that Lp05 does not merely follow the natural recovery trajectory but actively steers the ecosystem toward a more favorable composition. This dual action—boosting beneficial symbionts while restraining pathobionts—is a hallmark of genuine probiotic efficacy and has been documented for other lactobacilli strains in antibiotic-associated dysbiosis models (43–45).

The compositional shifts were accompanied by distinct changes in predicted metabolic functions. Notably, the placebo group exhibited a marked increase in the relative abundance of the lipoic acid metabolism pathway at S1, which persisted at S2. Lipoic acid is an essential cofactor for several key enzyme complexes in bacteria, and its metabolism has been linked to stress adaptation and antibiotic resistance in Proteobacteria (46, 47). The expansion of this pathway in the placebo group coincided with the bloom of Proteobacteria (mainly *Escherichia-Shigella*), suggesting that antibiotic*-*resistant opportunistic pathogens may exploit this metabolic route to gain a competitive advantage. Conversely, the Lp05 group showed a significantly lower abundance of the lipoic acid metabolism pathway at both S1 and S2, paralleling the suppression of Proteobacteria. Although PICRUSt2 predictions are hypothesis-generating rather than definitive, these observations raise the intriguing possibility that Lp05 may create a microenvironment less permissive for pathogen expansion by modulating the availability or utilization of key cofactors. Future studies employing metagenomic sequencing and targeted metabolomics are warranted to validate this mechanism.

Beyond metabolic modulation, *L. plantarum* strains are known to exert direct anti-pathogen effects through the production of organic acids, bacteriocins, and competitive exclusion (48). They also reinforce intestinal barrier integrity and modulate host immune responses via interaction with pattern recognition receptors (39, 49). While our study was not designed to dissect these molecular pathways, the observed enrichment of butyrate-producing genera (*Faecalibacterium*, *Blautia*, *Roseburia*) in the Lp05 group suggests a potential indirect benefit on short-chain fatty acid production and epithelial health (50–52). The concurrent increase in *Akkermansia muciniphila*, a mucin-degrading bacterium strongly associated with metabolic health and gut barrier function, further supports a global shift toward a “healthier” microbial consortium (53).

Although the relative abundances of Actinobacteria (0.7–4.5%) and Verrucomicrobiota (0.1–2.5%) observed in this study were low, these phyla harbor keystone species that exert disproportionately large effects on host physiology. This abundance range is consistent with previous reports in healthy populations, where Actinobacteria typically account for 0.8%–3.9% and Verrucomicrobiota range from 0.01% to over 10% depending on dietary and geographic factors (54, 55). Actinobacteria include the well-recognized probiotic genus *Bifidobacterium*, which contributes to gut barrier integrity and immune modulation, while Verrucomicrobiota are represented by *Akkermansia muciniphila*, a mucus-degrading bacterium strongly associated with metabolic health and gut barrier function (56, 57). The significant enrichment of these phyla in the Lp05 group at S2, despite their low absolute abundances, reflects a functionally meaningful shift in the gut ecosystem. This observation is consistent with the concept that keystone taxa, even at low abundance, can serve as important drivers of community structure and host health outcomes.

From a practical standpoint, the ability of Lp05 to reduce BQT-associated gastrointestinal adverse effects—without compromising eradication efficacy or safety—carries direct implications for patient management. Symptom burden is a major driver of poor adherence to *H. pylori* therapy, and incomplete eradication fuels antimicrobial resistance (58). By improving tolerability, Lp05 may indirectly contribute to higher real eradication success and help preserve the effectiveness of current first-line regimens. Moreover, the rapid recovery of a diverse, pathogen-suppressive microbiota after antibiotic exposure is likely to confer long-term benefits, including reduced risk of *Clostridioides* difficile infection and metabolic disorders (16). Given the excellent safety profile and low cost of probiotic supplementation, even modest clinical benefits support its integration into routine practice, particularly for patients with pre-existing gastrointestinal symptoms or those at high risk of dysbiosis (59, 60).

Regarding the observed fluctuations in serum lipids, it is important to emphasize that most values remained within the normal reference ranges throughout the study, and the number of patients with clinically significant dyslipidemia in the two groups is not statistically significant. The slight decrease in total cholesterol within the Lp05 group, while statistically significant, is of uncertain clinical relevance given its small magnitude and the fact that baseline cholesterol was higher in this group than in the placebo group. Similarly, the transient between-group differences in triglycerides likely reflect baseline imbalance rather than a true treatment effect. These lipid changes were not accompanied by any alterations in liver function tests or other metabolic parameters, further supporting the safety of Lp05. Nevertheless, the potential for probiotic strains to modulate host lipid metabolism has been reported in some studies, and our findings warrant further investigation in larger cohorts with longer follow-up, particularly in patients with baseline dyslipidemia (61, 62).

This study has several limitations. First, the sample size was calculated based on the expected improvement in gastrointestinal symptoms, not on the eradication rate. Consequently, the study was underpowered to detect a statistically significant difference in the primary endpoint, despite a clear positive trend. Larger, multi-center trials are needed to confirm whether the numerical increase in eradication rate represents a true effect. Second, the intervention period was relatively short, and long-term follow-up beyond six weeks was not performed. Whether the microbiota benefits persist or translate into reduced incidence of post-eradication metabolic or infectious diseases remains unknown. Third, because Lp05 was administered only as an adjunct to BQT and not as a standalone intervention, we cannot assess its independent therapeutic contribution in the absence of antibiotics. Future studies incorporating a probiotic-only arm or a preventive administration design would help disentangle the direct effects of Lp05 from those mediated through mitigation of antibiotic injury. Finally, the use of 16S rRNA amplicon sequencing limits taxonomic resolution and precludes functional confirmation at the gene or metabolite level. Multi-omics approaches—including shotgun metagenomics, metatranscriptomics, and metabolomics—are essential to elucidate the causal mechanisms underlying the observed phenotypic changes.

In conclusion, adjunctive *L. plantarum* Lp05 during bismuth quadruple therapy for *H. pylori* infection is safe, well tolerated, and provides significant clinical benefits by alleviating gastrointestinal symptoms and mitigating antibiotic-induced gut dysbiosis. While it does not significantly enhance eradication efficacy, it actively shapes a healthier and more resilient gut microbiota, characterized by enrichment of beneficial taxa and suppression of opportunistic pathogens. These effects are distinguishable from spontaneous post-antibiotic recovery and are supported by both compositional and functional signatures. Our findings position Lp05 as a valuable supportive strategy in *H. pylori* management, particularly for patients suffering from treatment-related side effects or those with heightened vulnerability to dysbiosis. Future research should focus on mechanistic elucidation and long-term health outcomes to further strengthen the evidence base for probiotic-assisted eradication therapy.

## Data Availability

The gut microbiota 16S rRNA sequencing data generated in this study have been deposited in the National Center for Biotechnology Information Sequence Read Archive under BioProject PRJNA1356691.

## CLINICAL TRIALS

Chinese Clinical Trials Registry, ChiCTR2400079562. URL: https://www.chictr.org.cn/showproj.html?proj=216368.
